# Cytogenetic studies in the redtail catfish, *Phractocephalus hemioliopterus* (Bloch & Schneider, 1801) (Siluriformes, Pimelodidae) a giant fish from Amazon basin

**DOI:** 10.3897/CompCytogen.v11i1.11152

**Published:** 2017-03-06

**Authors:** Ana Claudia Swarça, Ana Lucia Dias, Alberto Sergio Fenocchio

**Affiliations:** 1 Departamento de Histologia, CCB, Universidade Estadual de Londrina. 86051-970, Caixa Postal 6001, Londrina, Paraná, Brazil; 2 Departamento de Biologia Geral, CCB, Universidade Estadual de Londrina, Caixa Postal 6001, Londrina, Paraná, Brazil; 3 Departamento de Genética, Universidad Nacional de Misiones. Instituto de Biología Subtropical (IBS UNaM-CONICET). Félix de Azara 1552. 3300, Posadas, Misiones, Argentina

**Keywords:** Neotropical fish, Parrot catfish, karyotype, Ag-NORs, 18S rDNA, CMA_3_, C-banding

## Abstract

The objective of this study was to cytogenetically analyze *Phractocephalus hemioliopterus* comparing the findings with other data to infer relationships among Pimelodidae species. The results revealed a diploid number of 2n = 56 and the karyotype composed of 16 metacentric, 20 submetacentric, 6 subtelocentric and 14 acrocentric chromosomes (FN = 98). The Ag-NORs, 18S rDNA and CMA_3_ signals were coincident in location occupying the short arm of an acrocentric chromosome pair (23^th^), in a secondary constriction. The 5S rDNA genes were localized near the centromere on the short arms of one submetacentric chromosome pair. C-bands were localized predominantly in the terminal regions of chromosomes, including the AgNORs and a small metacentric pair with a conspicuous positive band on interstitial region. This chromosome pair could be considered a species-specific cytogenetic marker.

## Introduction

The genus *Phractocephalus* Agassiz, 1829 belongs to Pimelodidae family and contains three species, one extant, *Phractocephalus hemioliopterus* (Bloch & Schneider, 1801) and two extinct species recently described, *Phractocephalus nassi* (Lundberg and Aguilera, 2003) and *Phractocephalus acreornatus* (Aguilera et al. 2008). According to Lundberg et al. (1998) “isolation of peripheral drainage system south, west and north of the Paraná, Amazonas and Orinoco systems provided opportunity for allopatric divergence, and also was accompanied by much extirpation of once more widespread tropical fish species”.

The large catfish, *Phractocephalus hemioliopterus*, has a wide distribution in the lowland, meandering rivers and lagoons of the Orinoco, Amazon and Essequibo (Lundberg and Aguilera 2003) and as a monotypic taxon possesses several uniquely derived characteristics (de Pinna 1998). This catfish is known as “cajaro” in Venezuela and Colombia and in Brazil as “pirarara” (parrot - fish) because of its red or orange caudal fin (Lundberg and Aguilera 2003). In the areas of occurrence, the species has economic importance and is much appreciated by fishermen. However, in spite of its large size, *Phractocephalus* is also a common aquarium fish (Carvalho and Medeiros 2005).

From a systematic point of view, Pimelodidae remains as a controversial group, possessing some internal inconsistencies, represented by the “*Pimelodus* group”, “*Calophysus* group” and a basal branch including three genera *Phractocephalus*, *Leiarius* Bleeker, 1862 and *Perrunichthys* Schultz, 1944 (Lundberg and Littman 2003).

Available cytogenetic data partially support this hypothesis because several chromosomal studies on Pimelodidae have revealed that the species of this family have a predominant diploid number of 56 (Table 1) with a few exceptions, such as species included in the “*Calophysus* group” that show some characteristic cytogenetic features as 2n = 50, reported in *Calophysus* Müller & Trosche, 1843, *Luciopimelodus* Eigenmann & Eigenmann, 1888 and *Pinirampus* Bleeker, 1858 (Ramirez-Gil et al. 1998, Swarça et al. 1999, Sanchez et al. 2010) and *Megalonema platanum* (Günther, 1880) with 2n = 54 (Carvalho et al. 2011). According to phylogenetic tree of Lundberg and Littman (2003) the branch that includes *Leiarius*, *Perrunichthys* and *Phractocephalus* has been never studied cytogenetically; this fact demonstrates that more species must be chromosomally studied to increase the number of cytogenetic data to better understand the species relationships and the karyotypic evolution in this fish group. The present work aims to report for the first time the cytogenetic study of *Phractocephalus hemioliopterus*, a unique extant species of the genus *Phractocephalus* from the Amazon Basin.

**Table 1.** Cytogenetic data on the family Pimelodidae. 2n = diploid number. Only published data were used.

	2n	References
“*Pimelodus* group”		
*Bergiaria* Eigenmann & Norris, 1901	56	Dias and Foresti (1993)
*Iheringichthys* Eigenmann & Norris, 1900	56	Carvalho et al. (2004); Carvalho and Dias (2005); Carvalho et al. (2010); Vissotto et al. (1999); Ribeiro et al. (2008); Sanchez et al. (2014)
*Parapimelodus* La Monte, 1933	56	Treco et al., 2008.
*Pimelodus* Lacepède, 1803	Predominant 56	Schell (1973); Toledo and Ferrari (1976); Dias and Foresti (1993); Vissotto et al. (1999); Swarça et al. (2001b); Borin and Martins-Santos (2002); Souza et al. (2003); Borin and Martins-Santos (2004); Souza et al. (2004a, b); Garcia and Moreira Filho (2005); Treco and Dias (2009); Moraes-Neto et al. (2011)
“*Calophysus* group”		
*Calophysus* Müller & Troschel, 1843	50	Ramirez-Gil et al. (1998)
*Pinirampus* Bleeker 1858	50	Swarça et al. (1999); Sanchez et al. (2010)
*Luciopimelodus* Eigenmann & Eigenmann, 1888	50	Sanchez et al. (2010)
“*Megalonema* group”		
*Megalonema* Eigenmann, 1912	54	Carvalho et al. (2011)
“*Sorubiminae* group”		
*Hemisorubim* Bleeker, 1862	56	Martins-Santos et al. (1996); Swarça et al. (2013)
*Pseudoplatystoma* Bleeker, 1862	56	Fenocchio and Bertollo (1992); Martins-Santos et al. (1996); Swarça et al. (2005b), Moraes-Neto et al. (2011); Nirchio et al. 2013
*Zungaro* Bleeker, 1858	56	Martins-Santos et al. (1996); Swarça et al. (2001c)
*Sorubim* Cuvier, 1829	56	Fenocchio and Bertollo (1992); Martins-Santos et al. (1996); Moraes-Neto et al. 2011
*Brachyplatystoma* Bleeker, 1862	56	Gonçalves et al. (2014)
*Steindachneridion* Eigenmann & Eigenmann, 1919	56	Swarça et al. (2005a); Swarça et al. (2006); Moraes-Neto et al. (2011)
*Phractocephalus* Agassiz, 1829	56	Present data

## Material and methods

Six specimens of *Phractocephalus hemioliopterus* from Amazon Basin/Brazil maintained in the fishing farm of the Universidade Estadual de Londrina were studied cytogenetically. The chromosome preparations were obtained from lymphocyte culture according to Fenocchio and Bertollo (1988), avoiding the sacrifice of specimens. Silver staining of NORs (AgNORs) was performed using the method of Howell and Black (1980). C banding and Chromomycin A_3_ (CMA_3_) staining were carried out using the methods of Sumner (1972) and Verma and Babu (1995), respectively. Fluorescence *in situ* hybridization (FISH) experiments were performed using biotinylated 18S rDNA probes (1700 bp fragments) obtained from the nuclear DNA of the fish *Oreochromis niloticus* (Linnaeus, 1758) labeled with biotin-14-dATP by nick translation (Gibco cat Nº 18247-015), according to the manufacturer’s instructions. The hybridization technique, post-hybridization washes and visualization were carried out following Swarça et al. (2001c). The preparations were analyzed in an Olympus BX50 microscope, and the best metaphases were captured with a SONY camera, model Exware HAD coupled to the microscope. The FISH slides were observed and the images acquired with a Leica DM 4500 microscope equipped with a DFC 300F9 camera and Leica IM50 4.0 software. Chromosome morphology was determined on the basis of Levan et al. (1964) and Guerra (1986) with some modifications and chromosomes were classified as metacentric (m), submetacentric (sm), subtelocentric (st) and acrocentric (a). NF (chromosome arm number) was determined considering m/sm/st chromosomes having two arms and acrocentric chromosomes having one arm.

## Results and discussion

The family Pimelodidae is composed of 109 valid species (Eschmeyer and Fong 2016), but only 27 species have been analyzed cytogenetically (Swarça et al. 2007). *Phractocephalus* is a monotypic genus. The only species of the genus, *Phractocephalus hemioliopterus*, is widely distributed in the rivers of the Orinoco, Amazon and Essequibo basins (Lundberg and Aguilera 2003). The extinction of the other two species (*Phractocephalus nassi* and *Phractocephalus acreornatus*) was hypothetically explained by Lundberg et al. (1998).

**Figure 1.**
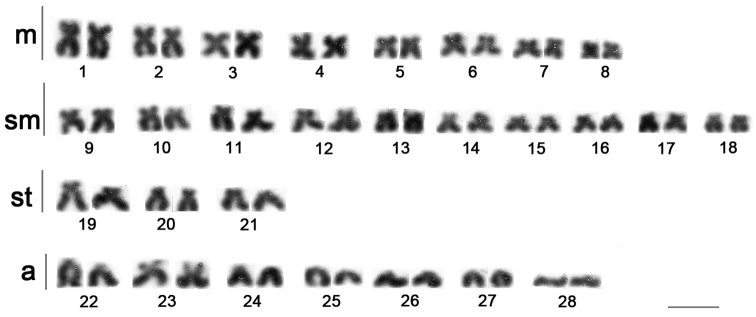
Karyotype of *Phractocephalus hemioliopterus*. Conventional Giemsa staining. Scale bar: 5 µm.

The diploid number (2n = 56) and karyotype constitution, 16m, 20sm, 6st, 14a (FN = 98) of *Phractocephalus hemioliopterus* is reported for the first time (Fig. 1). According to Swarça et al. (2000) the chromosome number is identical to other large species that belong to the “*Pimelodus* group” comprising at least *Hemisorubim* Bleeker, 1862, *Zungaro* Bleeker, 1858, *Sorubim* Cuvier, 1829, *Pseudoplatystoma* Bleeker, 1862 that could be called informally “Sorubiminae group” and includes the largest catfishes from South America (de Pinna 1998, Lundberg and Littman 2003) (Table 1). Although *Phractocephalus hemioliopterus* does not belong to these systematic and/or taxonomic groups, this species shares many cytogentic traits, such as the chromosome shape, size and staining patterns, with the species included in “Sorubiminae”.

**Figure 2.**
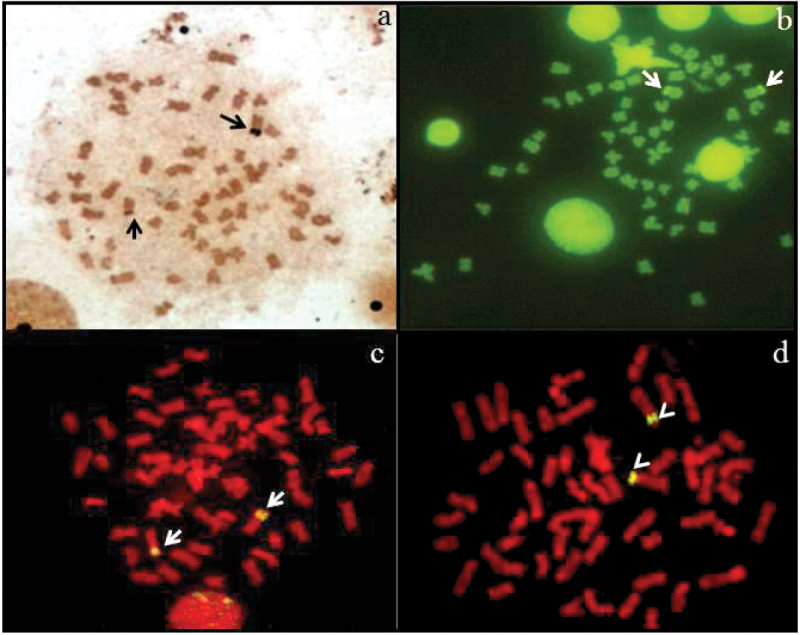
Metaphases of *Phractocephalus hemioliopterus* **a** AgNO_3_ staining **b** CMA_3_ banding **c** FISH with 18S rDNA probe and **d** FISH with 5S rDNA probe. Arrows indicate the NOR-bearing chromosomes and arrowheads indicate the chromosome pair with 5S rDNA.

As stated below, diploid number 56 with high fundamental number, NORs located at the terminal position on the short arm of an acrocentric chromosome pair (23^th^), coincident with positive C-bands (Fig. 2a) represent common features in almost all pimelodid species analyzed so far, suggesting that these cytogenetic traits were conserved during the karyotype evolution and may have an ancient common origin (Sanchez et al. 2010). The presence of ribosomal genes on the short arm of one st/a chromosome pair is coincident with the location observed in the “*Calophysus* group” (Sánchez et al. 2010) and “Sorubiminae group” (Swarça et al. 2008) and differs from the “*Pimelodus* group”, where the ribosomal genes are located almost exclusively on the long arm of m/sm chromosome pairs (Swarça et al. 2007).

The data obtained with CMA_3_ indicate that the Ag-NORs of *Phractocephalus hemioliopterus* are rich in GC pairs (Fig. 2b), a general pattern also found in the family Pimelodidae by several authors (Swarça et al. 2001a, b, c, Garcia and Moreira-Filho 2005, Swarça et al. 2005b, Nirchio et al. 2013, among others). However, the exact location of ribosomal genes on chromosomes could be revealed exclusively by means of *in situ* hybridization using 18S and 5S rDNA probes. After application of this procedure these regions showed bright signals on short arms of one subtelocentric pair (18S rDNA probe) and on short arms of another submetacentric chromosome pair (5S rDNA probe) (Fig. 2c, d). In general, the 18S and 5S rDNA sites are not syntenic but located on different chromosome pairs, this feature being the most frequent patter in several Pimelodidae species (Carvalho et al. 2010, Swarça et al. 2008, 2009). However, recently syntenic localization of the major rDNA clusters and the 5S sites were reported in other species (Ziemniczak et al. 2012, Konerat et al. 2014, da Rocha et al. 2016). So far, both patterns of rDNA and 5S rDNA localization, syntenic and not syntenic, have been described in Pimelodidae. Still, the evolutionary trend of ribosomal genes chromosome distribution has not been yet outlined.

**Figure 3.**
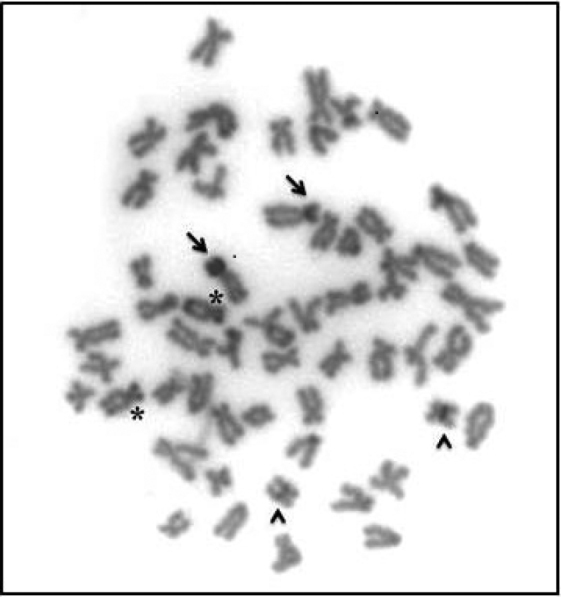
Somatic metaphase of *Phractocephalus hemioliopterus* after C-banding. Arrowheads indicate the chromosome pair with interstitial heterochromatin; arrows indicate the NOR-bearing chromosome and asterisks indicate the chromosomes with heterochromatin blocks in both terminal regions.

Heterochromatin distribution revealed by C-banding was evidenced on telomeric regions of some chromosomes, one pair with bitelomeric bands and in the secondary constriction on the short arm of NOR bearing pair (Fig. 3). This last feature represents a common trait shared by most pimelodids. Another interesting cytogenetic characteristic is the presence of a small metacentric pair that shows a conspicuous heterochromatic block in interstitial region (Fig. 3). Heterochromatin interstitially located has been reported in some species of the family Pimelodidae, such as *Pseudoplatystoma tigrinum* (Valenciennes, 1840) (Fenocchio and Bertollo 1992), *Hemisorubim platyrhynchos* (Valenciennes, 1840) (Martins-Santos et al. 1996), *Iheringichthys labrosus* (Lütken, 1874) (Vissotto et al. 1999) and also in species of the genus *Pimelodus* Lacépède, 1803 (Treco et al. 2008). The interstitial localization of a strong C-band in *Phractocephalus hemioliopterus* on a small metacentric chromosome can be a species-specific cytogenetic marker and could be useful for future studies on the internal relationships of the species included in this group.

Taking into consideration the findings described previously, the present work is the first to provide cytogenetic information about *Phractocephalus hemioliopterus*

The cytogenetic description of *Phractocephalus hemioliopterus* allowed the karyotypic characterization and the comparison of certain cytogenetic features shared in general with other Pimelodidae, however, some of these traits distinguish the “Sorubiminae group”, suggesting that this species could be integrated into the branch of the great catfishes.
